# Discordant Gender Identity and Surgical Goals in Two Adults with Comparable Ambiguous Genitalia: Two Case Reports from a Resource-Limited Setting

**DOI:** 10.7759/cureus.111019

**Published:** 2026-06-17

**Authors:** Brenda Ainomugisha, Rogers Kajabwangu, Onesmus Byamukama, Kalyebara P Kato, Verena Geissbuhler, Musa Kayondo

**Affiliations:** 1 Obstetrics and Gynecology, Mbarara University of Science and Technology, Mbarara, UGA; 2 Urogynecology, University Hospital of Basel, Basel, CHE

**Keywords:** ambiguous genitalia, clitoromegaly, differences of sex development, dysgerminoma, gender identity

## Abstract

Disorders of sex development (DSD) are rare congenital conditions characterized by discordance among chromosomal, gonadal, and phenotypic sex. Adult presentation of ambiguous genitalia is not uncommon, particularly in low-resource settings where delayed diagnosis, limited access to specialized care, sociocultural stigma, and financial barriers constrain evaluation and management. We present two adults with remarkably comparable external genital appearances but profoundly different gender identities, social roles, treatment interests, and therapeutic goals.

The two patients, both aged 25 years, presented to our institution with comparable ambiguous external genitalia but different gender roles and concerns. Case one is a phenotypic female presenting with an enlarged clitoris and partially obstructed vaginal introitus, causing her severe psychosocial distress and inability to engage in sexual intercourse. She had normal female secondary sexual characteristics and regular menstruation. Imaging demonstrated normal internal female reproductive organs. Due to financial limitations, karyotyping was not performed. She desired vaginal opening and removal of the penile-like structure. She underwent examination under anesthesia, introital reconstruction, and clitoral reduction surgery, with good postoperative functional and psychological outcomes. Case two is an individual raised and socially recognized as male who presented with progressive abdominal distension and chronic pelvic pain. Despite a masculine gender identity and virilized phenotype, the patient reported menstruation since adolescence. Imaging demonstrated multiple abdominal masses suspicious for malignancy. He was desirous of the removal of the uterus to eliminate menstruation, which was a major source of his distress, irrespective of whether he would sexually perform as a male or not. Exploratory laparotomy revealed large intra-abdominal tumors, and histopathology confirmed dysgerminoma. The patient is currently undergoing chemotherapy.

These cases highlight the complexity of adult DSD presentation in resource-constrained settings and underscore the importance of individualized, patient-centered management that prioritizes gender identity, psychosocial well-being, reproductive goals, and informed consent. The report further emphasizes the ethical and clinical challenges posed by limited diagnostic capacity and delayed presentation.

## Introduction

Ambiguous genitalia, a condition in which the appearance of the external genitalia don't conform to the typical male or female, is a challenging presentation [1]. It is most commonly recognized in the neonatal period, though some individuals remain undiagnosed until adolescence or adulthood, particularly in low-resource settings where specialized diagnostic services are unavailable and sociocultural stigma discourages healthcare seeking [2, 3]. This is a common manifestation of disorders of sex development (DSD), a heterogeneous group of congenital conditions characterized by atypical development of chromosomal, gonadal, or anatomical sex [4].

Normal sexual differentiation depends on a highly coordinated sequence of genetic, hormonal, and embryological events [5]. Risk factors for DSD are multifactorial and include genetic, chromosomal, hormonal, environmental, and familial factors [6]. Chromosomal abnormalities such as Turner syndrome (45, X), Klinefelter syndrome (47, XXY), and sex chromosome mosaicism interfere with normal gonadal development [7]. Others include a positive family history of DSD, infertility, unexplained neonatal deaths, or genital anomalies and maternal exposure during pregnancy to androgenic medications, progestins, endocrine-disrupting chemicals, or androgen-producing tumors [8].

The estimated global prevalence of clinically significant DSD is approximately 1 in 4500-5500 live births [9]. Accurate diagnosis of DSD requires a multidisciplinary approach involving detailed clinical assessment, hormonal evaluation, karyotyping, imaging, and increasingly, molecular genetic testing [10]. While these advances have substantially improved DSD care in high-income countries, delayed diagnosis and limited access to specialized services remain common in many low- and middle-income countries [10-13]. DSD also has profound implications for psychological well-being, gender identity, sexual function, fertility, social integration, and quality of life [11]. Contemporary management has therefore shifted from a solely anatomy-based approach toward patient-centered care that emphasizes informed decision-making, psychosocial support, preservation of sexual function, and respect for individual gender identity and autonomy [14]. Furthermore, certain DSD conditions are associated with an increased risk of gonadal malignancy, including dysgerminoma and gonadoblastoma, making timely diagnosis and surveillance essential components of care [15, 16].

We present two adults with remarkably comparable external genital anatomy suggestive of ambiguous genitalia but who exhibited profoundly different gender identities, life experiences, and healthcare goals. While one patient identified as female and sought genital reconstruction to facilitate sexual function, the other lived socially as male and presented primarily with a large intra-abdominal dysgerminoma. These cases illustrate the complexity of DSD management in resource-constrained settings and underscore the importance of individualized, patient-centered care that extends beyond anatomical considerations alone.

## Case presentation

Case one

A 25-year-old nulliparous woman presented with concerns regarding an enlarged phallus-like clitoris and a nearly closed vaginal opening despite identifying and living as female. She reported the onset of menarche at 13 years of age, regular menstrual cycles, normal breast development, and otherwise typical female secondary sexual characteristics. She had never engaged in penetrative sexual intercourse because of fear and embarrassment related to her genital appearance, despite having meaningful intimate relationships and a desire for sexual activity. The patient had completed tertiary education but was unemployed and residing with an aunt following the death of both parents. She reported never having disclosed her genital condition to anyone, including close relatives, and described years of emotional distress and social withdrawal before finally seeking medical care. There was no reported history of childhood illness, genital trauma, or exposure to exogenous androgens.

**Figure 1:**
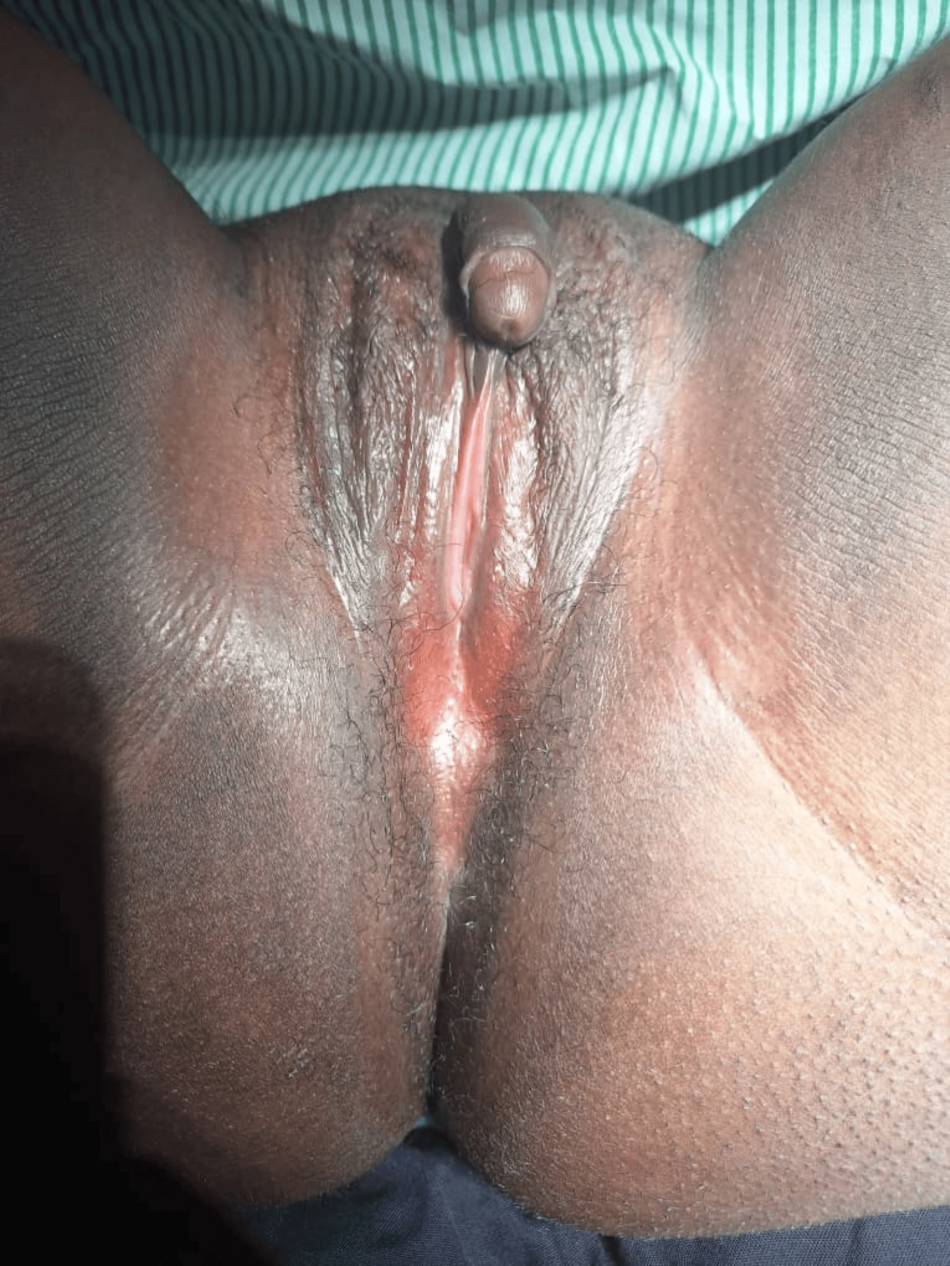
Case one before surgery

On general examination, she appeared phenotypically female, measuring approximately 188 cm in height, with well-developed breasts, smooth skin, absence of facial hair, and a feminine voice. Her vital signs were stable, with blood pressure of 111/71 mmHg, pulse rate of 76 beats/minute, temperature of 36.7°C, and weight of 74 kg. Systemic examination was unremarkable. Perineal examination, as shown in Figure 1, revealed marked clitoromegaly measuring approximately 5 cm in length and 2 cm in width without a urethral opening. Inferior to the enlarged clitoris was a thin longitudinal membrane measuring approximately 3 cm with a small distal circular opening. Beneath this opening, a vaginal depression was palpable posterior to the perineal skin. The anal opening was normally positioned approximately 4 cm distally within the same longitudinal plane. The staging on the Prader scale was two.

A provisional diagnosis of ambiguous genitalia with suspected 46, XX DSD was made. Pelvic ultrasonography demonstrated a normal uterus, cervix, and right ovary. A cystic lesion measuring 33 × 4 × 20 mm with multiple internal echoes and a thick wall was identified within the left ovary. No abnormalities were detected in the urinary tract or upper abdominal organs. Due to financial limitations, karyotyping and hormonal assays could not be performed. The patient expressed a strong desire for surgical correction of the vaginal obstruction and removal of the enlarged clitoris after extensive counseling regarding anatomy, sexual function, and potential risks of surgery.

Surgical Procedure

**Figure 2:**
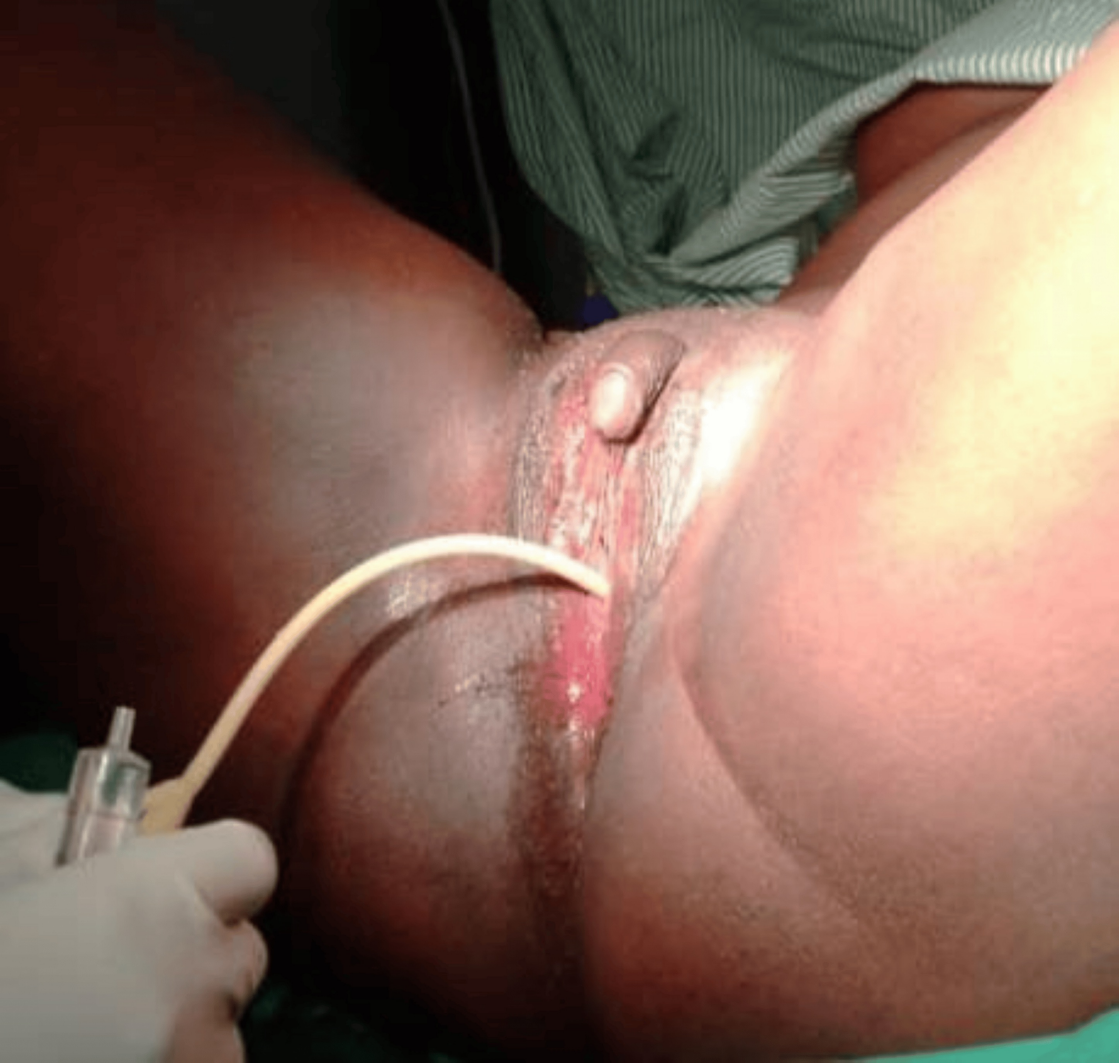
Case one catheterized

Under spinal anesthesia and lithotomy positioning, examination under anesthesia confirmed a normal urethral opening located superior to the vaginal dimple. Catheterization, as shown in Figure 2, verified that the enlarged clitoris was not associated with the urinary tract. A diamond-shaped incision was created at the vaginal opening to enlarge the introitus. A vaginal canal approximately 6 cm long and 2 cm wide was identified, and the cervix was visualized. Vaginal mucosa was mobilized and anchored to the labial and perineal skin edges. A 20-mL syringe barrel was inserted and secured as a temporary vaginal stent. Clitoral reduction was subsequently performed by excision at the base near the mons pubis, followed by reconstruction and layered closure with meticulous hemostasis. Postoperatively, the patient remained hospitalized for 10 days and received antibiotics, analgesics, and perineal hygiene care. The urinary catheter and vaginal stent were removed on postoperative day seven, after which progressive vaginal dilation using Hegar dilators and topical estrogen cream was initiated.

**Figure 3:**
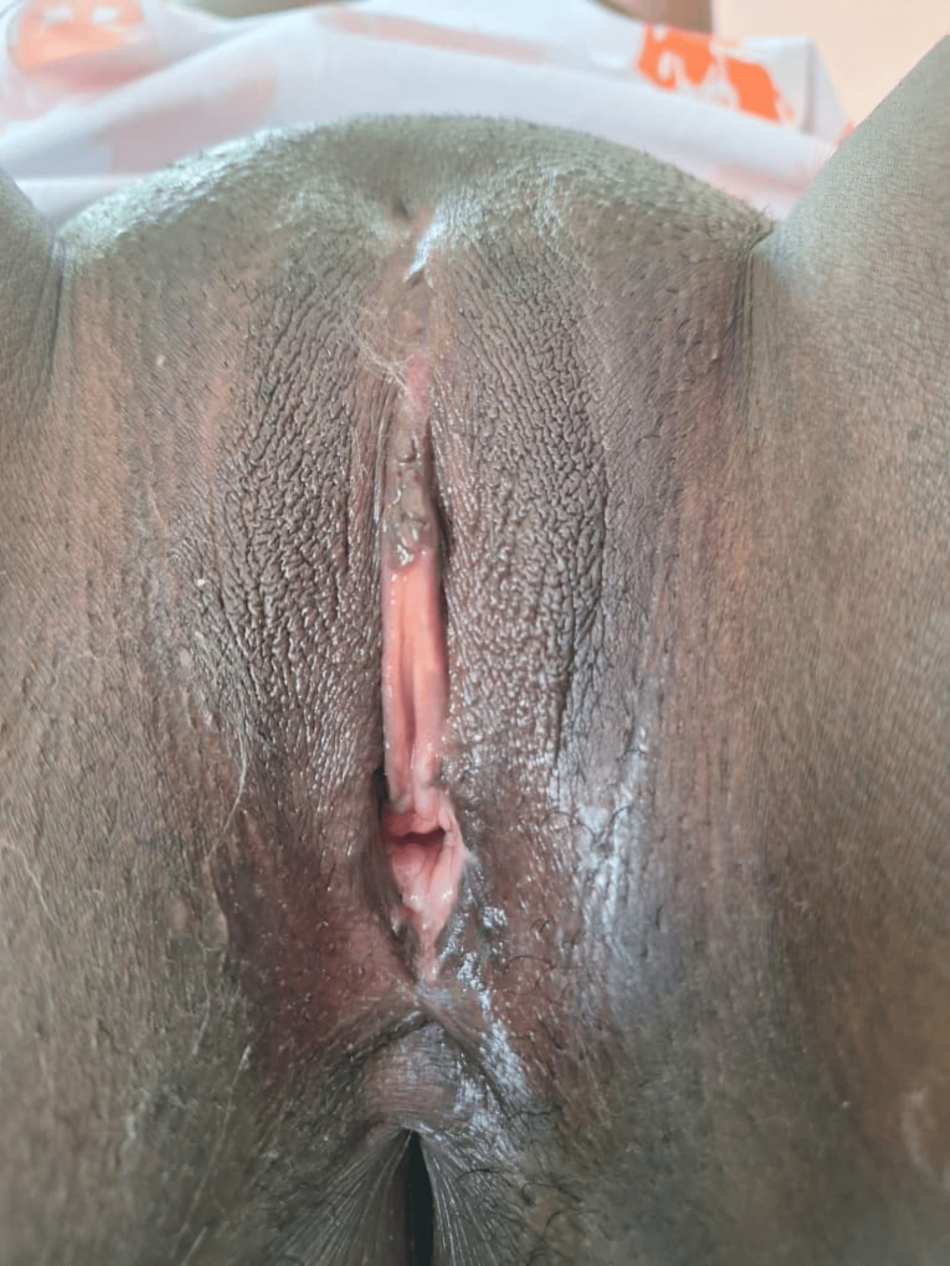
Case one six weeks post-surgery

At the six-week follow-up, the patient reported marked improvement in psychological well-being and satisfaction with the surgical outcome. Examination demonstrated a well-healed perineum and a functional vaginal canal measuring approximately 7 cm in length and 3 cm in width, as shown in Figure 3. She was also reviewed at three months and six months postoperatively, and she was happily sexually active without a complaint. Physical examination showed a long wide vaginal opening of about 7 cm long by 3 cm wide.

Case two

A 25-year-old individual raised and socially recognized as male presented with progressive abdominal distension and lower abdominal pain of one year duration. During clinical evaluation, the patient disclosed the onset of virilization during adolescence, including the development of facial hair and a deepened voice, but also reported regular menstruation beginning at 15 years of age. The patient had been assigned male gender at birth because of the presence of a phallus, which had enlarged progressively with age. However, urine did not pass through the phallus. The patient identified socially as male and expressed a desire for relief from the abdominal swelling and cessation of menstruation. Both parents were deceased, and the patient was accompanied by a community health worker in whom he had previously confided. He had no formal education and survived on casual labor.

On examination, the patient appeared chronically ill and wasted but demonstrated masculine body habitus, facial hair, a hoarse voice, smooth skin, and well-developed breasts. Vital signs were stable. Abdominal examination revealed gross distension with marked suprapubic tenderness. A large solid pelvic mass measuring approximately 18 × 10 cm was palpable, in addition to another irregular mass within the left lumbar region. Bilateral inguinal nodular swellings were also noted. Perineal examination demonstrated a phallus measuring approximately 6 × 3 cm without a urethral opening. A closed introitus with a small distal dimple was observed, through which urine was passed with straining in the lithotomy position. The stage, according to the Prader score, was 2. The anal opening was normally positioned. A provisional diagnosis of DSD complicated by gonadal malignancy was made. 

Investigations

Abdominal ultrasonography demonstrated four large hypervascular heterogeneous intra-abdominal masses located in the left lumbar region, rectovesical pouch, epigastrium, and right inguinal canal. The uterus could not be clearly visualized. Computed tomography of the abdomen and pelvis revealed two large complex abdominal masses displacing pelvic organs, including the uterus and urinary bladder. As in the first case, karyotyping, hormonal studies, and tumor markers (lactate dehydrogenase (LDH), alpha fetoprotein, and serum beta human chorionic gonadotropin (HCG)) could not be performed because of financial constraints.

Surgical Procedure

Exploratory laparotomy through a midline abdominal incision revealed a massive intra-abdominal tumor with a dense capsule adherent to bowel, mesentery, and uterus, occupying nearly the entire abdominal cavity. Careful adhesiolysis was performed, and the tumor was excised together with the left fallopian tube. A second mesenteric mass adherent to the transverse colon was also identified and excised. The liver appeared grossly normal. The uterus was visualized and appeared morphologically normal, although both ovaries could not be clearly identified from the pelvic masses intraoperatively. The clinical staging at laparotomy was IIIC. The abdomen was closed in layers, and tissue specimens were submitted for histopathological evaluation.

**Figure 4:**
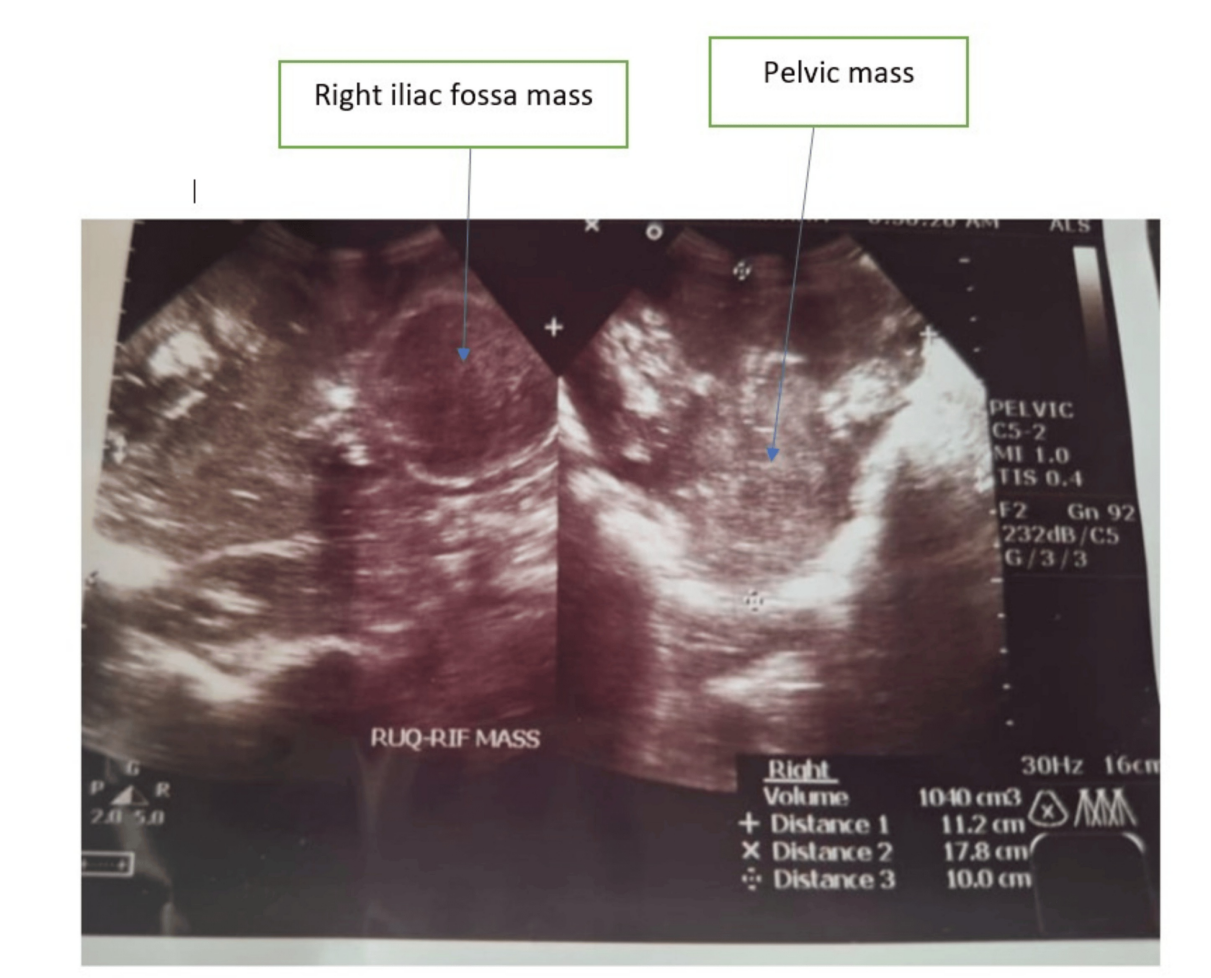
Case two abdominal ultrasound scan

**Figure 5:**
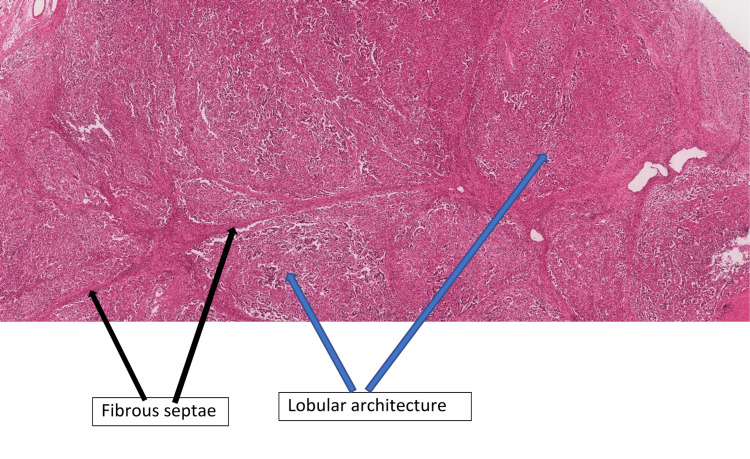
Case two histology image showing the fibrous septae, and lobular architecture typical of dysgerminoma Magnification X 100

**Figure 6:**
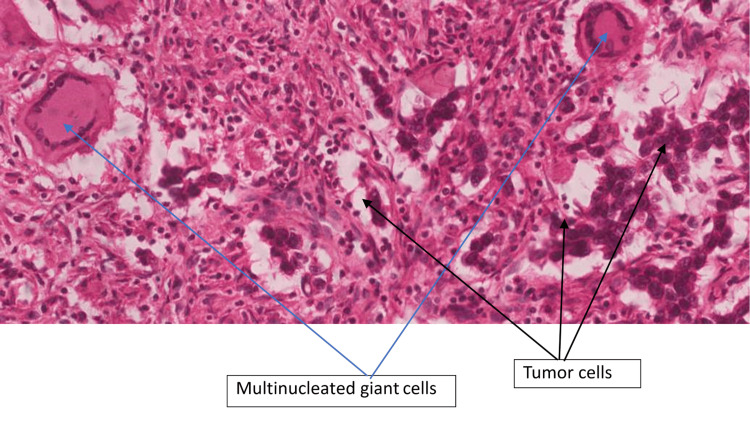
Case two histology image showing the tumor cells and giant cells found in dysgerminoma Magnification X 200

**Figure 7:**
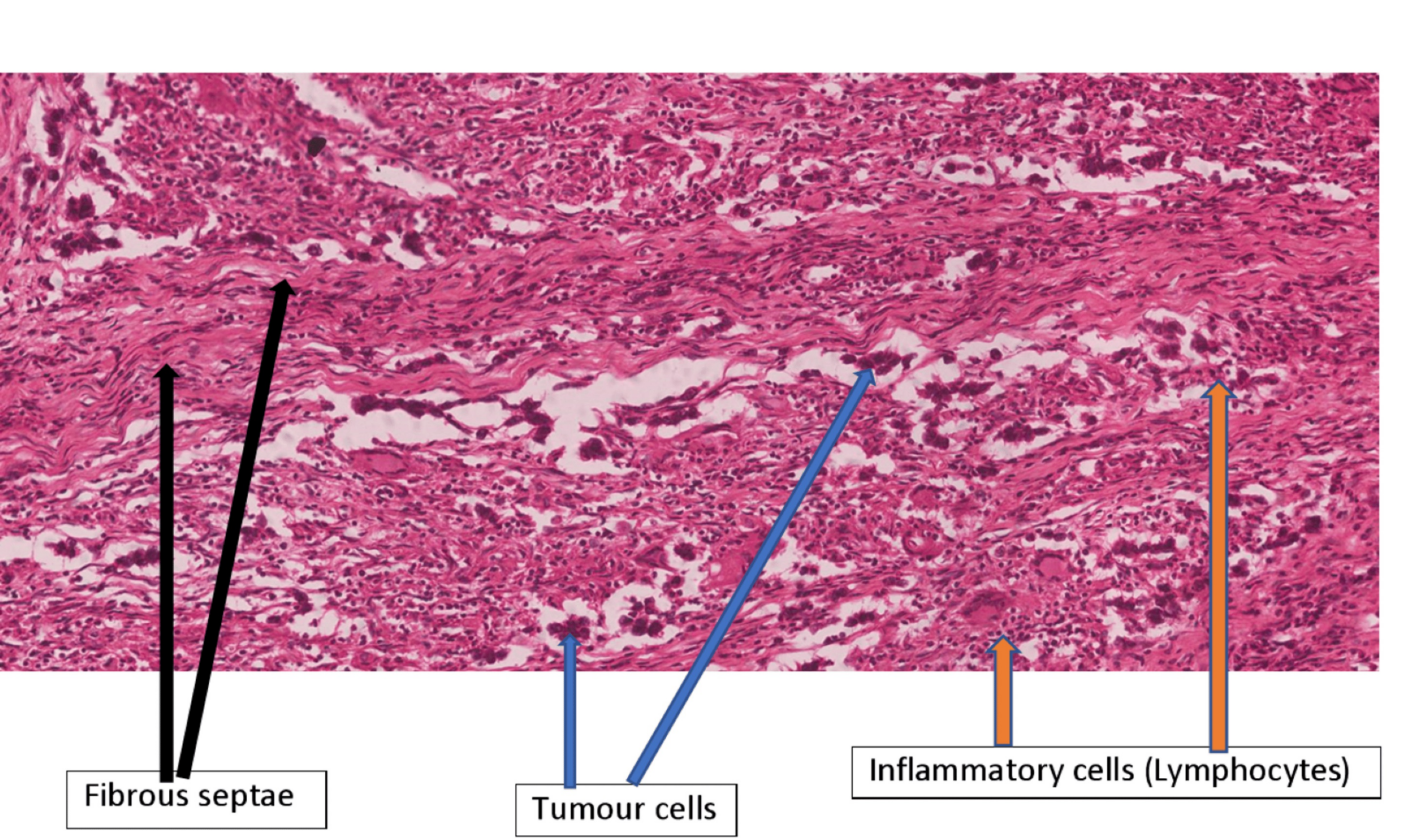
Case two histology image showing the fibrous septae, tumor cells, and lymphocytes found in dysgerminoma Magnification X 50

The postoperative recovery was uneventful. Histopathology confirmed dysgerminoma. The patient was subsequently referred to gynecologic oncology services for further management and is currently undergoing chemotherapy (bleomycin, etoposide, and cisplatin (BEP)). Figure 4 shows the abdominal masses on ultrasound scan, while Figures 5-7 show the histology slide typical of dysgerminoma with the fibrous septae, lobular architecture, giant cells, lymphocytes, and typical tumor cells under Hematoxylin and Eosin (H&E) stain.

These two cases have remarkable similarities but also differences. Table 1 below displays the comparison of the two cases along the clinical characteristics, gender identity, investigations, and management. Table 2 displays the shared and divergent features among the two cases.

**Table 1:** Comparison of clinical characteristics, gender identity, investigations, and management of the cases

Characteristic	Case one	Case two
Age at presentation	25 years	25 years
Reason for presentation	Removal of the penile-like structure and vaginal reconstruction for sexual function	Treatment of the abdominal masses
Phenotypic presentation	Ambiguous genitalia	Ambiguous genitalia
Gender identity	Female	Male
Gender role/social upbringing	Lived socially as female	Lived socially as male
Psychosexual concerns	Sexual function and body image	Gender affirmation, cessation of menstruation, and treatment of abdominal masses
Hormonal evaluation	Not done due to financial constraints	Not done due to financial constraints
Karyotype testing	Unavailable	Unavailable
Tumor markers	Not applicable	Not done due to financial constraints
Imaging findings	Ovaries and uterus were present	Large intra-abdominal masses and uterus visualized
Histology findings	Not applicable	Dysgerminoma
Surgical management	Genital reconstructive surgery	Tumor excision

**Table 2:** Shared and divergent features between the two cases DSD - disorders of sex development

Shared features
Ambiguous genitalia
Delayed diagnosis
Resource-limited setting
Need for multidisciplinary care
Potential DSD diagnosis
Divergent features
Gender identity
Social gender role
Treatment priorities
Surgical goals and procedures done
Presence of gonadal malignancy

## Discussion

These two cases illustrate the extraordinary heterogeneity of DSD presentation and emphasize that external genital appearance alone does not predict gender identity, psychosocial adaptation, or therapeutic priorities [5, 17]. Despite remarkably comparable phenotypic findings, the patients had fundamentally different social identities, expectations, and goals of care.

The first patient identified strongly as female and sought restoration of vaginal function to facilitate sexual intimacy and improve body image. Her presentation highlights the severe psychosocial burden experienced by many adults living with untreated ambiguous genitalia. Shame, fear of rejection, social isolation, and anxiety regarding intimate relationships are frequently reported among individuals with DSD, particularly in settings where cultural stigma limits disclosure and access to care [18, 19]. The second patient, in contrast, had lived socially as male despite menstruation and the presence of internal female reproductive structures. His principal concerns were relief of symptoms related to abdominal swelling and cessation of menses rather than genital reconstruction. This discordance between anatomy and gender identity underscores the importance of individualized management and avoidance of assumptions based solely on phenotype [5].

Clitoromegaly may occur in a variety of DSD conditions, including congenital adrenal hyperplasia, ovotesticular DSD, and androgen excess states [20]. Although contemporary approaches emphasize preservation of sexual sensation and caution regarding clitoral surgery, adult patients who independently request genital reconstruction after informed counseling may benefit significantly in terms of psychosocial well-being and sexual confidence [21]. In the first case, the patient's postoperative satisfaction and improved quality of life suggest that carefully individualized surgical intervention can be beneficial. The second case additionally highlights the oncologic risks associated with DSD. Dysgerminoma is the ovarian counterpart of seminoma and is among the most common malignant germ cell tumors encountered in individuals with gonadal dysgenesis [16, 22, 23]. Delayed diagnosis may result in advanced intra-abdominal disease, as observed in case two.

Ethical considerations are central to DSD management [24]. Historically, irreversible surgical procedures were frequently performed in infancy without patient involvement. Modern practice increasingly recognizes patient autonomy and informed decision-making as essential components of care [25, 26]. Both patients in this report actively participated in treatment decisions, and management plans were largely aligned with their expressed identities and personal goals.

Although definitive etiological classification may not be possible without endocrine and genetic investigations, a systematic clinical assessment can allow identification of individuals with DSD, guide sex assignment decisions, and enable appropriate management and counseling of patients and families [17]. In many low-resource settings, this pragmatic approach remains the foundation of DSD diagnosis and management until advanced diagnostic services become available [27, 28]. Earlier diagnosis, psychosocial counseling, multidisciplinary evaluation, and affordable genetic testing could substantially improve the current management and long-term outcomes for affected individuals.

## Conclusions

Adults with DSD may present with comparable anatomical findings but profoundly different gender identities, psychosocial experiences, and treatment priorities. Management should therefore be individualized, patient-centered, and grounded in informed consent. These cases demonstrate the complexity of DSD care in low-resource settings and highlight the importance of respecting patient autonomy while addressing functional, psychological, and oncologic concerns. We acknowledge the limitation of our inability to conduct karyotyping, hormonal assessment, and tumour markers for definitive diagnosis and management.
